# Unraveling the Conundrum: A Rare Case of Mucinous Cystadenoma in the Uterus

**DOI:** 10.7759/cureus.89387

**Published:** 2025-08-05

**Authors:** Mohammed Imaduddin, Datla Swati Priya, Vinay Mamidala, Mohammed Owais, Muddusetty Muralidhar

**Affiliations:** 1 Department of Surgical Oncology, CION Cancer Clinics, Hyderabad, IND; 2 Department of Pathology, Unipath Specialty Laboratory Limited, Hyderabad, IND; 3 Department of Medical Oncology, CION Cancer Clinics, Hyderabad, IND

**Keywords:** mucinous cystic neoplasm, mucinous ovarian cancer, mucinous tumors, ovarian tumors, uterine neoplasms/therapy

## Abstract

Extra-ovarian recurrence of mucinous cystadenomas (MCs) is a rare phenomenon. This case report presents the first documented instance of a benign MC recurring within the uterine myometrium of a 48-year-old woman. Two years following an oophorectomy for a large left ovarian MC, the patient presented with lower abdominal pain. Imaging revealed a pelvic mass and subsequent surgery identified a cystic lesion arising from the uterine fundus. Histopathological examination confirmed a benign MC. This case underscores the importance of considering a broad differential diagnosis for pelvic cystic lesions, especially in patients with a history of mucinous tumors. It highlights the potential diagnostic challenge posed by such a rare presentation and emphasizes the need for further research into mechanisms underlying extra-ovarian recurrences.

## Introduction

Mucinous cystadenomas (MCs) are epithelial tumors predominantly arising from the ovaries, constituting a significant portion of benign ovarian neoplasms [1]. These tumors are characterized by cystic structures lined with mucin-producing epithelial cells. While the vast majority of MCs are ovarian in origin, documented cases of extra-ovarian occurrences, albeit rare, exist. Reported sites for these extra-ovarian MCs include the appendix, mesentery, and uterine isthmocele [2,3].

The potential for recurrence of benign MCs, although uncommon, presents a significant clinical concern due to its implications for patient management and prognosis. Recurrence can occur at the original ovarian site or can involve distant dissemination within the abdominal cavity. Several factors are known to contribute to the risk of recurrence, including incomplete surgical excision, intraoperative spillage, the presence of microscopic residual disease following surgery, and potential genetic predispositions. Additionally, the initial size of the tumor, specific histopathological features, and the presence of any borderline or frankly invasive components can influence the likelihood of recurrence [4].

This case report presents a unique and, to our knowledge, the first documented instance of an MC recurring within an unusual location, the uterine myometrium. We discuss the clinical presentation, diagnostic workup, and surgical management of this case. Furthermore, we explore the established risk factors for MC recurrence and delve into the potential mechanisms underlying this rare presentation. This case highlights the importance of considering a broader differential diagnosis when encountering pelvic cystic lesions, particularly in patients with a history of mucinous tumors.

## Case presentation

A 48-year-old premenopausal woman presented with a six-month history of lower abdominal pain, described as dragging in character. She denied any other concerning symptoms. Two years prior, she had undergone a left salpingo-oophorectomy at another center for a large (17 x 15 cm) left ovarian MC. A review of the surgical notes revealed an intraoperative cyst rupture with spillage of gelatinous content.

**Figure 1:**
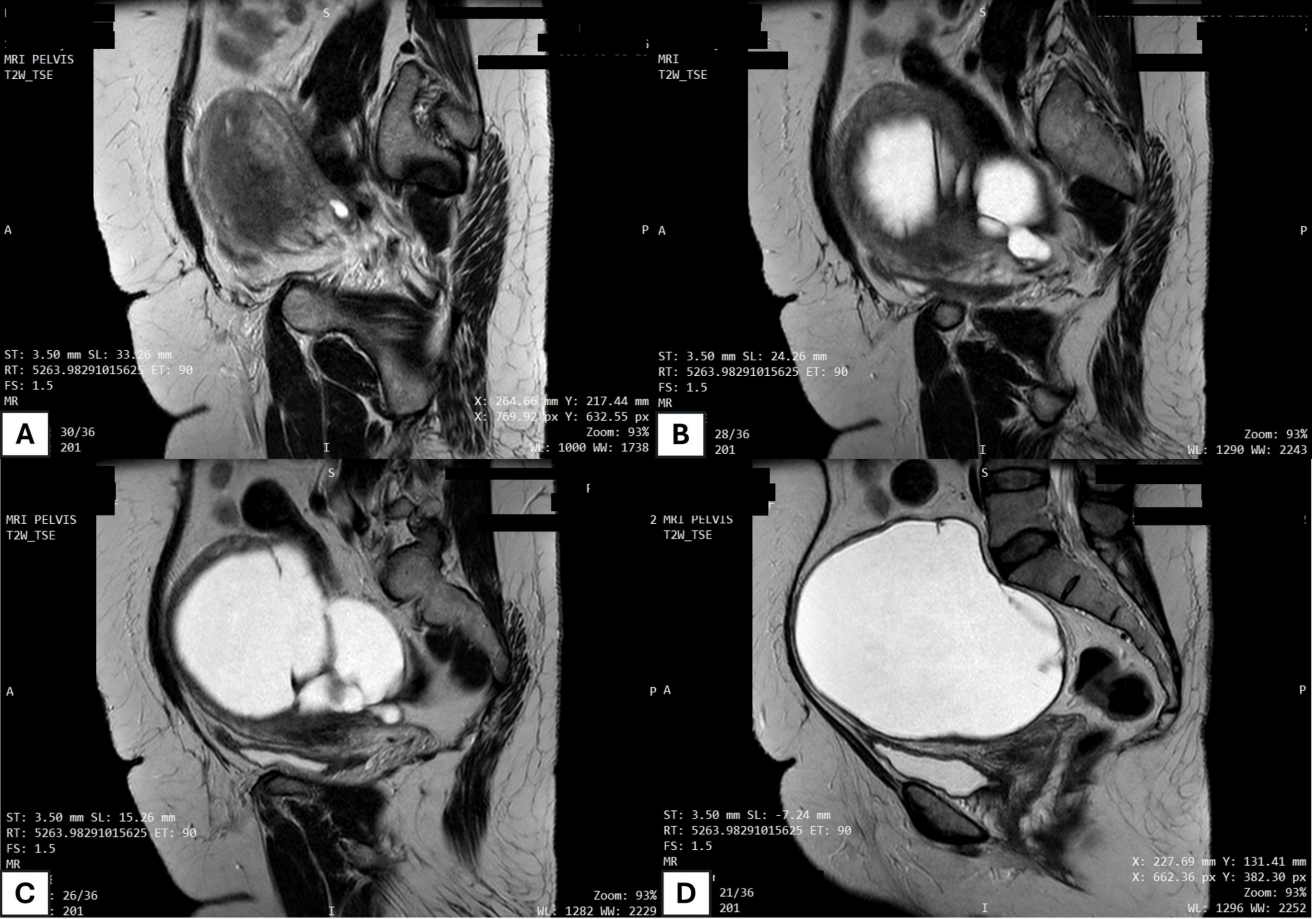
MRI scan showing the sagittal sections of the pelvis (A-D) Lateral to medial sections showing a large cystic lesion in the pelvis, possibly arising from the uterus. MRI: magnetic resonance imaging

On examination, a pelvic mass was palpable in the lower abdomen. An MRI scan of the pelvis revealed a lobulated multicystic lesion with multiple thick internal septations arising from the left side of the pelvis. The lesion demonstrated a broad interface with the serosal surface of the left lateral uterine wall, suggesting a possible uterine origin (Figure 1).

**Figure 2:**
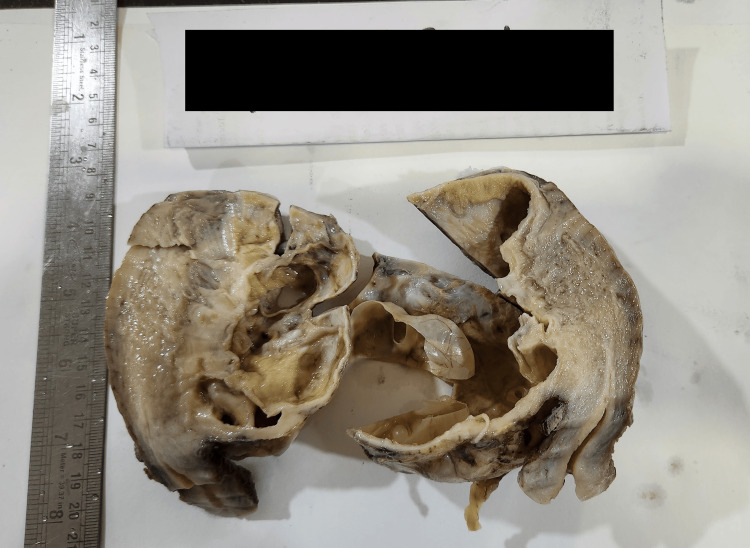
Gross image of the specimen Myometrium shows a large cystic cavity with internal loculations. Cut section shows a smooth inner wall and a multi-loculated cyst.

Serum CA-125 and CEA levels were within normal limits. Following informed consent, the patient underwent surgery. Intraoperative findings revealed a large cystic lesion arising from the posterior body and fundus of the uterus. A total abdominal hysterectomy with right salpingo-oophorectomy was performed. Gross examination demonstrated a large (12 x 10 x 9 cm) cystic cavity within the uterine myometrium (Figure 2).

**Figure 3:**
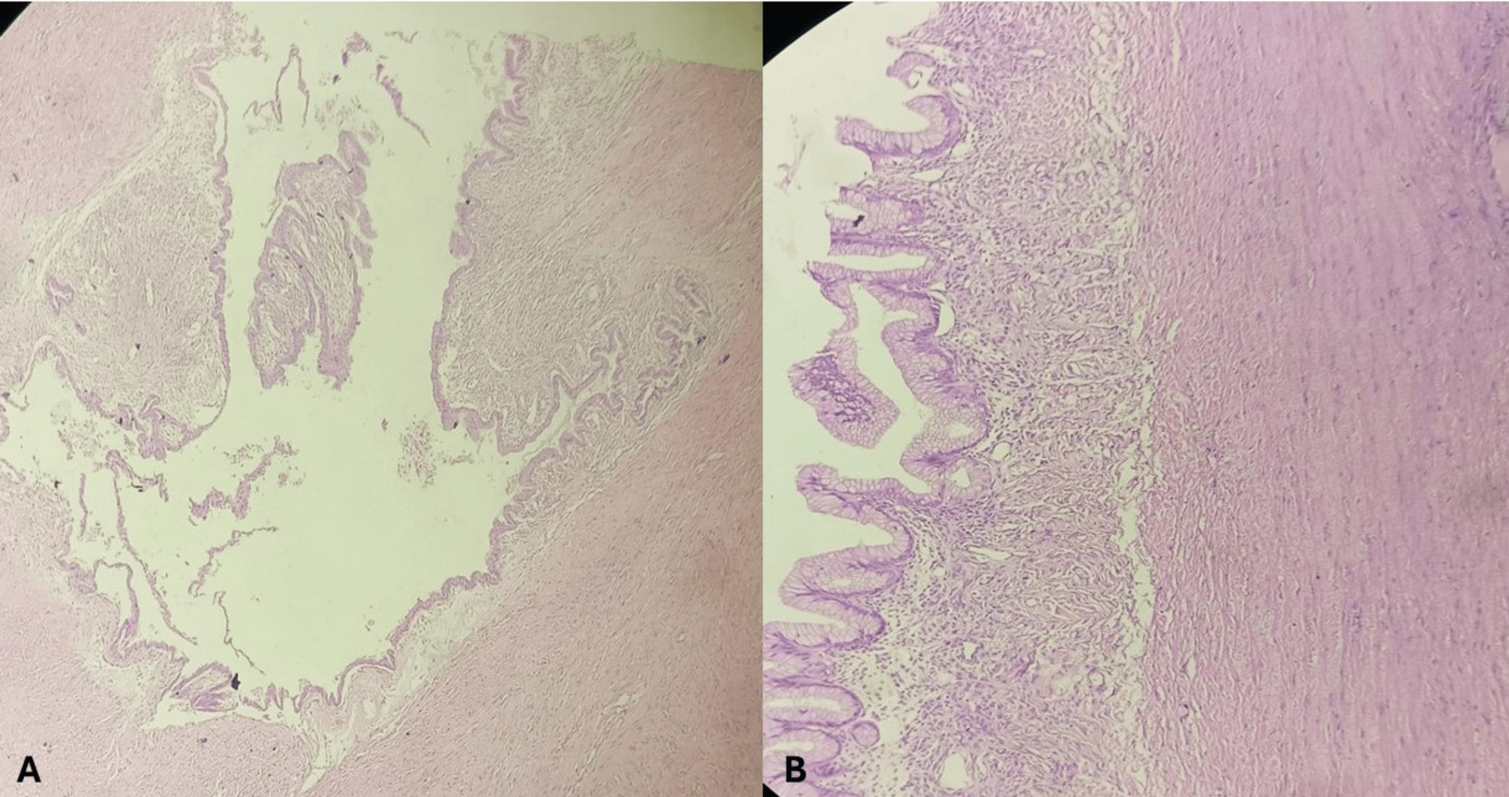
Microscopic image (A) 20x image of the mucinous cyst wall in the myometrium of the uterus. (B) 40x image of the mucinous lining of the cyst wall with the ovarian stroma, lying over the underlying smooth muscle bundles of the myometrium.

The cut surface revealed a smooth inner wall and multiloculated compartments filled with mucinous material. The lesion was located away from the remaining ovary and fallopian tube. Microscopic examination confirmed a single-layered, tall columnar mucinous epithelium lining the cystic cavity, devoid of atypia. The cyst was seen lying over smooth muscle bundles of myometrium (Figure 3).

Based on these pathological findings, the diagnosis of a benign MC arising from the myometrial wall was established. The patient recovered well postoperatively and remains asymptomatic at her six-month follow-up.

## Discussion

This case report presents a unique instance of a benign MC recurring within the uterine myometrium of a 48-year-old woman. To our knowledge, this is the first reported case of such a recurrence site. MCs are most commonly benign epithelial tumors arising from the ovary. While recurrence is uncommon, it can occur at the original site or spread to distant locations. Established risk factors for recurrence include incomplete surgical excision, presence of microscopic residual disease, potential genetic predispositions, larger initial tumor size, and specific histopathological features [4].

The prior surgical history in this case raises suspicion for potential contributing factors to the unusual recurrence. The initial surgery involved an open salpingo-oophorectomy with reported intraoperative cyst rupture. Cyst rupture during surgery is a documented risk factor for increased recurrence rates, possibly due to implantation of mucinous cells at the site of spillage [5].

The current recurrence within the uterine myometrium poses an interesting diagnostic challenge. MCs arising from the uterus itself are extremely rare, with only a handful of case reports documented in the literature, primarily involving the cervix or endometrium [6]. The differential diagnosis in this case would have likely included other possibilities, such as a metastatic mucinous carcinoma or an endometriotic cyst. Cystic adenomatoid tumors of the uterus were another differential considered. The presence of mucin-producing epithelium and the absence of a central solid component or hobnail-like epithelium argue against a cystic adenomatoid tumor [7]. Ultimately, the histopathological findings confirmed the diagnosis of a benign MC with no evidence of malignancy.

The exact mechanism underlying the myometrial recurrence in this case remains unclear. Possible explanations include mucinous metaplasia of the endometrial or endocervical glands with subsequent cyst formation within the myometrium; implantation of spilled mucinous cells during the initial surgery into the myometrium, with subsequent growth; or undiagnosed residual microscopic foci of mucinous epithelium from the original ovarian tumor inadvertently left behind during surgery, which subsequently grew within the myometrium. While the definitive cause cannot be ascertained in this specific case, it highlights the importance of considering a broader differential diagnosis when encountering pelvic cystic lesions, especially in patients with a history of mucinous tumors.

Finally, this unique presentation emphasizes the need for further research to elucidate the mechanisms underlying extra-ovarian and myometrial recurrences of MCs. This knowledge can guide the development of improved surgical techniques and potentially inform future treatment strategies.

## Conclusions

This case highlights the importance of maintaining a high index of suspicion for recurrent MCs, even in unusual locations like the uterine myometrium. A comprehensive clinical evaluation, meticulous surgical exploration, and thorough histopathological examination are crucial for establishing the correct diagnosis and guiding appropriate management. Further research is warranted to elucidate the exact mechanisms underlying such rare presentations of extra-ovarian and myometrial recurrences of MCs.
